# Can Medical Herbs Stimulate Regeneration or Neuroprotection and Treat Neuropathic Pain in Chemotherapy-Induced Peripheral Neuropathy?

**DOI:** 10.1155/2013/423713

**Published:** 2013-07-31

**Authors:** Sven Schröder, Kathrin Beckmann, Giovanna Franconi, Gesa Meyer-Hamme, Thomas Friedemann, Henry Johannes Greten, Matthias Rostock, Thomas Efferth

**Affiliations:** ^1^HanseMerkur Center for Traditional Chinese Medicine at the University Medical Center Hamburg-Eppendorf, Martinistraße 52, 20246 Hamburg, Germany; ^2^ICBAS, University of Porto, Rua de Jorge Viterbo Ferreira No. 228, 4050-313 Porto, Portugal; ^3^Department of Systems Medicine, Tor Vergata University, 00133 Rome, Italy; ^4^Hubertus Wald Tumorzentrum, University Cancer Center Hamburg, University Medical Center Hamburg-Eppendorf, Martinistraße 52, 20246 Hamburg, Germany; ^5^Department of Pharmaceutical Biology, Institute of Pharmacy and Biochemistry, Johannes Gutenberg University, Staudinger Weg 5, 55128 Mainz, Germany

## Abstract

Chemotherapy-induced neuropathy (CIPN) has a relevant impact on the quality of life of cancer patients. There are no curative conventional treatments, so further options have to be investigated. We conducted a systematic review in English and Chinese language databases to illuminate the role of medical herbs. 26 relevant studies on 5 single herbs, one extract, one receptor-agonist, and 8 combinations of herbs were identified focusing on the single herbs *Acorus calamus rhizoma*, *Cannabis sativa fructus*, *Chamomilla matricaria*, *Ginkgo biloba*, *Salvia officinalis*, *Sweet bee venom*, *Fritillaria cirrhosae bulbus,* and the herbal combinations Bu Yang Huan Wu, modified Bu Yang Huan Wu plus Liuwei Di Huang, modified Chai Hu Long Gu Mu Li Wan, *Geranii herba* plus *Aconiti lateralis praeparata radix* , Niu Che Sen Qi Wan (Goshajinkigan), Gui Zhi Jia Shu Fu Tang (Keishikajutsubuto), Huang Qi Wu Wu Tang (Ogikeishigomotsuto), and Shao Yao Gan Cao Tang *(Shakuyakukanzoto)*. The knowledge of mechanism of action is still limited, the quality of clinical trials needs further improvement, and studies have not yielded enough evidence to establish a standard practice, but a lot of promising substances have been identified. While CIPN has multiple mechanisms of neuronal degeneration, a combination of herbs or substances might deal with multiple targets for the aim of neuroprotection or neuroregeneration in CIPN.

## 1. Introduction

Several chemotherapeutic drugs are known to be neurotoxic and can lead to chemotherapy-induced peripheral neuropathy (CIPN). It is one of the main dose-limiting toxicities in oncologic treatments and a potential reason to terminate or suspend chemotherapy, in some cases leading to disease progression [1]. CIPN involves damage to the peripheral nervous system and can produce severe neuropathic pain [2, 3], sensory deficits, or gait impairment [4] and can severely decrease the patient's quality of life [5]. Sensory symptoms usually develop before motor symptoms, because motor neurons are more myelinated [6, 7]. Distal parts of the axons are the first affected, so sensory symptoms typically start symmetrically and bilaterally from the tips of the toes and fingers and progress proximally in a “stocking-glove” distribution [8]. The incidence of CIPN can reach levels of up to 92% [9]. The major groups of drugs that induce CIPN include the antitubulins (paclitaxel, docetaxel, ixabepilone, and vincristine), platinum analogs (cisplatin, carboplatin, and oxaliplatin), and the proteasome inhibitors bortezomib and thalidomide [1]. Patients with a preexisting other cause of peripheral neuropathy develop more severe and persistent CIPN [10–12].

The development of CIPN is usually dependent on the cumulative dose and symptoms may progressively aggravate [10]. After sustaining therapy symptoms are usually reversible, but in some cases they may be irreversible [10, 13] and sometimes even progress after stopping medication, especially after treatment with vinca alkaloids (e.g., vincristine), platinum analogues (e.g., cisplatin and oxaliplatin), and taxanes (e.g., paclitaxel) [14, 15]. Mostly the sensory nerve cell bodies of the dorsal root ganglia are affected, or the afferent and efferent distal peripheral axons are damaged [16].

Some differences concerning the mechanisms of CIPN are described for different therapeutic drugs. 

In platinum compounds the dorsal root ganglia are the main parts of the nervous systems that are injured [17] and apoptosis is induced via structural alterations of DNA and of cell-cycle kinetics [18], triggered by oxidative stress and mitochondrial dysfunction but possibly downregulated by a reduction of enzymes like p53 [19–22]. 

The taxanes paxlitaxel and docetaxel are antitubulins and mainly damage the soma of the sensory neurons and the nerve axons. This is induced by an interference with the axonal transport, which is caused by enhancement of microtubule polymerization [23]. Microglial activation in the spinal cord and high concentration in the dorsal root ganglia tissue induce CIPN [24]. Like platinum compounds, taxanes can damage dorsal root ganglia. This is induced by macrophage activation in the dorsal root ganglia but occurs also in the peripheral nerve [24]. Paclitaxel induces a massive polar reconfiguration of axonal microtubules and an impairment of organelle transport [25]. 

Vinca alkaloids (e.g., vincristine) prevent tubulin polymerization from soluble dimers into microtubules [26]. By affecting the tubulin dimers loss of axonal microtubules and alterations in their length, arrangement and orientation are produced [27, 28]. This alters the neuronal cytoskeleton, leading to abnormalities in axonal transport and axonal degeneration [28, 29]. Decreasing affinity for tubulin of the vinca alkaloids explains the different neurotoxicity profiles and the severity of CIPN [30].

Epothilones (e.g., ixabepilone) are like the taxanes antitubulins, so there might be similar mechanisms of peripheral neurotoxicity. They damage the ganglion soma cells and peripheral axons through disruption of microtubules of the mitotic spindle and interfere with the axonal transport in the neurons [31] and can also induce polymerization of tubulin dimers in microtubules. Additionally they stabilize preformed microtubules against conditions favouring de-polymerization [32, 33].

Bortezomib might induce pathological changes in Schwann cells and myelin, axonal degeneration, and dorsal root ganglia neuron changes [34, 35], as well as chromatolysis of dorsal root ganglial neurons. It causes cytoplasmic accumulation of eosinophilic material [36] and interferes with the transcription, nuclear processing and transport, as well as with the cytoplasmic translation of messenger RNAs in dorsal root ganglions [37]. Mitochondrial and endoplasmic reticulum-mediated calcium dysregulation plays an important part [38, 39]. The activation of the mitochondria-based apoptotic pathway or inhibition of the transcription of the nerve growth factor by interference with the nuclear factor-*κ*B pathway can lead to disarrangement of the neurotrophin network [39, 40]. Bortezomib can induce changes in all major primary afferent fibres [41].

The structure of thalidomide is characterized by a 3-substituted glutarimide ring and a ph-thalimide ring, which are sensitive to enzymatic or nonenzymatic hydrolysis [42], but despite several studies it has not been possible to identify the responsible enzymes for the production of neurotoxic metabolites, so its mechanism of peripheral neurotoxicity is still elusive [43].

Multiple drugs have been tested, mainly in animal studies, for their putative neuroprotective activity in CIPN. A few components which can protect different tissues from toxic agents were clinically tested, showing conflicting results. No conclusive reports confirming their effectiveness have been provided [44, 45] and a reduction of anticancer activity was suspected [46]. Several neurotrophins were tested but there was no evidence of neuroprotection [47–50]. Erythropoietin, a multifunctional trophic factor, showed promising results in preclinical studies [51, 52] but has relevant safety problems [53].

Antioxidants have been tested as neuroprotectants [54–59], but no conclusive evidence of neuroprotection has yet been found.

Neuropathic pain is conventionally treated by antiepileptic and tricyclic antidepressant drugs, but these drugs are ineffective for treatment of decreased sensation and cannot induce neuroprotection or neuroregeneration. Other compounds with different mechanisms (e.g., acetyl-L-carnitine, glutamate carboxypeptidase II inhibitors, calpain inhibitors, a solution of calcium and magnesium) have now been investigated in preclinical stages with unknown value for clinical routine [59–63].

Thus specific and effective curative treatments for CIPN are lacking, especially those meant for enhancing neuroregeneration or neuroprotection [64–67], and the evaluation of further treatment options is of great importance. While the effectiveness of herbal treatment is not well known yet, this review was done to illuminate the actual and potential future role of herbal treatment in CIPN.

## 2. Methods

To review the existing clinical and experimental studies of herbal treatment in CIPN, a systematic literature search was performed from the databases from inception up until January 2013 using MEDLINE, Google Scholar, Cochrane Database, CINHAL (Cumulative Index to Nursing and Allied Health Literature), CNKI (China National Knowledge Infrastructure), and Wanfang Med Online and ISI Proceedings for conference abstracts. The keywords searched were as follows: 

(Chinese herbs or herbs or plants or Chinese medicine as MeSH term) AND (neuropathy or chemotherapy). The CINHAL, CNKI, and Wanfang Med Online Databases did not allow logical searches with AND, so we used simple combinations of the search words. Historical searches of reference lists of relevant articles were also undertaken.

To be included in our review a study had to focus on the topic CIPN and neuropathy in human and animal models irrespective of design. Papers with at least an English abstract were included. Study selection was performed by two reviewers (SSch and KB) with disagreement resolved by discussion and adjudication. 

Listed articles concerning diseases other than CIPN and herbal treatment were excluded, while animal products used in the tradition of herbal medicine were included. 

## 3. Results

A total of 3474 (1477 in English and 1997 in Chinese databases) articles were retrieved by way of electronic searches and examination of reference lists of clinical and review articles. After screening titles and/or abstracts, 3376 articles were excluded because either the focus was on an intervention other than CIPN and herbal treatment or they were duplicated studies or not relevant. From a total of 98 articles which were retrieved for detailed evaluation, 15 were included in the review, focusing on 5 single herbs, one extract, one receptor agonist, and 8 combinations of herbs. For a summary of the experimental and clinical studies see Table 1.

### 3.1. Single Herbs or Single Herbal Compounds

#### 3.1.1. **Acorus calamus rhizoma**

This herb (family: Araceae) is traditionally used in the treatment and management of pain and severe inflammatory in Ayurveda. It is commonly used to relieve the muscle, joint, vascular and nerve injury associated with severe inflammatory and neuropathic pain [68]. In a rat model a hydroalcoholic extract of *Acorus calamus rhizoma *has been shown to exert beneficial effect on neuropathic pain induced by tibial and sural nerve transection [69]. In a further study *Acorus calamus rhizoma* extract attenuated sciatic nerve chronic constriction injury and induced ameliorated behavioral (hyperalgesia and allodynia), biochemical (superoxide anion, myeloperoxidase, and total calcium), and histopathological (axonal degeneration) changes [68]. Another study investigated the protective effect of *Acorus calamus rhizoma* extract in vincristine-induced painful neuropathy. Hydroalcoholic extracts of *Acorus calamus rhizoma *attenuated vincristine-induced behavioral and biochemical changes to an extent comparable to pregabalin (positive control) and attenuated vincristine-induced painful neuropathy, which probably may be attributed to its multiple effects including antioxidative, anti-inflammatory, and calcium inhibitory activity [70].

#### 3.1.2. *Cannabis sativa*

Two structurally distinct cannabinoid CB2 agonists—the aminoalkylindole (R,S)-*AM1241* ((R,S)-(2-iodo-5-nitrophenyl)-[1-((1-methyl-piperidin-2-yl)methyl)-1H-indol-3-yl]-methanone) and the cannabilactone *AM1714* (1,9-dihydroxy-3-(1′,1′-dimethylheptyl)-6H-benzo[c]chromene-6-one))—had been tested for their dose related suppression of established paclitaxel-evoked mechanical allodynia in a rat model. *(R)-AM1241*, but not *(S)-AM1241*, suppressed paclitaxel evoked mechanical allodynia, and *AM1714* induced a modest antinociceptive effect. So the authors suggested that cannabinoid CB2 receptors may be important therapeutic targets for the treatment of chemotherapy-evoked neuropathy [71]. *Cannabis sativa* or *Cannabis* extracts have not been clinically explicitly tested for CIPN but for other clinical conditions like neuropathic pain in HIV [72]. Multiple clinical trials examined the effect on neuropathic pain and found positive effects on central and peripheral neuropathic pain with different forms of application [73–82].

#### 3.1.3. *Matricaria chamomilla*

This is a commonly used herb in western as well as in eastern phytopharmacological tradition. Flavonoids from *Matricaria chamomilla* seem to have an antispasmodic effect and main components such as *α*-bisabolol or chamazulene have anti-inflammatory effects [83]. En-In-Dicycloether has both antispasmodic and anti-inflammatory effects together [84]. In a mouse model it was shown that *Matricaria chamomilla extract-*treated mice had a significant reduction of cisplatin-induced peripheral pain [85]. *Matricaria chamomilla* hydroalcoholic extract was able to decrease cisplatin-induced pain and inflammation better than morphine [85].

#### 3.1.4. *Ginkgo biloba*

The popular herb from the maidenhair tree that has shown some promising effects as an neuroprotectant. The most unique components of the extracts are the terpene trilactones, that is, ginkgolides and bilobalide [109]. In vitro and ex vivo studies indicate that bilobalide has multiple mechanisms of action that may be associated with neuroprotection, including its preservation of mitochondrial ATP synthesis, its inhibition of apoptotic damage induced by staurosporine or by serum-free medium, its suppression of hypoxia-induced membrane deterioration in the brain, and its action in increasing the expression of the mitochondrial DNA-encoded COX III subunit of cytochrome C oxidase and the ND1 subunit of NADH dehydrogenase [110]. Because multiple modes of action may apply to bilobalide, it could be useful in developing therapy for neurodegeneration [109–111]. Oztürk et al. investigated *Ginkgo biloba* alcoholic extract in cisplatin-induced peripheral neuropathy in mice [86]. Development of neuropathy was evaluated with changes in sensory nerve conduction velocity (NCV) and *Ginkgo biloba* extract prevented reduction in NCV. In another study a *Ginkgo biloba* extract prevented some functional and morphological deteriorations induced by cisplatin, antagonizing the decrease in the number of migrating cells and in the length of outgrowing axons [86]. Marshall et al. investigated retrospectively 17 patients with colorectal cancer who received oxaliplatin along with *Ginkgo biloba *extract, but no specification of the extraction method was provided in the published abstract. The researchers found that 11 of the 17 patients developed a grade 1 peripheral neuropathy (PN) after the first cycle of oxaliplatin. Five of six patients who received *Ginkgo biloba* after the second cycle of oxaliplatin reported decreased intensity and duration of sensory PN. No *Ginkgo biloba* related side effects have been observed. The data suggested that *Ginkgo biloba* extract appears to attenuate the intensity and duration of acute dysesthesias caused by oxaliplatin and may yield synergistic antitumor activity [87].

#### 3.1.5. *Salvia officinalis*

*Salvia* species and their isolated constituents possess significant antioxidant activity in enzyme-dependent and enzyme-independent systems [112–115]. The flavonoid apigenin, for example, has been shown to protect neurons against A*β*-induced toxicity [116]. In addition to antioxidant activity, many *salvia* species and their isolated constituents showed anti-inflammatory properties [117, 118]. *Salvia officinalis* extract can have anti-inflammatory and also antinociceptive effects on chemical behavioral models of nociception in mice that involve an opioid mechanism [119]. An animal study showed the effects of the *Salvia officinalis* hydroalcoholic extract on vincristine-induced PN in mice in comparison with morphine with a decrease of pain response, suggesting that *Salvia officinalis* extract could be useful in the treatment of vincristine-induced peripheral neuropathic pain [88].

#### 3.1.6. *Sweet bee venom*

The venom of honey bees with its active peptide Melittin has been tested for injection into the acupuncture point Zusanli (ST 36) for its effect on CIPN in animal models. It showed to alleviate thermal hyperalgesia and mechanical allodynia. The results indicated an association with the activation of the LC noradrenergic system and with a reduction in spinal pNR1 [120, 121].

In a first case series this was tested in 5 patients in a 1-week course of treatment, which showed no side effects and gave evidence of clinical improvement [89]. Another prospective case series of this procedure analyzed the clinical observations made on 11 CIPN patients treated with *Sweet bee venom*. A total of 11 eligible consecutive CIPN patients were treated for 3 weeks and observed for another 3 weeks. A significant intraindividual improvement was found for pain and neuropathy scales [90]. 

#### 3.1.7. Verticinone from *Fritillaria bulbus*

Verticinone, an isosteroidal alkaloid isolated from *Fritillaria bulbus*, was evaluated in mice for its analgesic activities in murine models of inflammatory and neuropathic pain. It was shown that oral administration of hydroalcoholic extracted verticinone could significantly inhibit acetic acid-induced writhing response in a dose-dependent manner superior to acetylsalicyl acid. In the rat model of paclitaxel induced neuropathic pain, in contrast to the declined analgesic effect of morphine after repeated administration with the same dose, a relatively constant analgesic effect of verticinone was observed, so verticinone is expected to become a potentially novel sedative-analgesic agent without producing tolerance [91].

### 3.2. Herbal Combinations

#### 3.2.1. Bu Yang Huan Wu (Chin.) = Tonify the Yang to Restore Five-Tenths Decoction (Engl.)

*Bu Yang Huan Wu* is a classical combination of Chinese herbs, first mentioned in Wang Qing-Ren's *Yi Lin Gai Cuo* (Correcting the Errors in the Field of Medicine) published in 1830 [122]. This recipe contains *Astragalus membranaceus radix, Angelica sinensis radix, Prunus persicae semen, Paeoniae rubra radix, Ligustici chuanxiong rhizoma, Lumbricus terrestris, Spatholobi caulis, Curcuma radix, Chaenomeles lagenaria fructus*, and* Achyranthes bidentatae radix*. 

The decoction has been used in a randomized Chinese study of 84 patients (treatment group *n* = 44, control group *n* = 40) after the treatment of oxaliplatin and showed reduced development of CIPN in the treatment group tested by standardized clinical tests [92]. 

#### 3.2.2. Modified Bu Yang Huan Wu (Chin.) = Tonify the Yang to Restore Five-Tenths Decoction (Engl.) plus Liuwei Di Huang (Chin.) = Rokumijiogan (Jap.) = Pilula Rehmannia Sex Saporum (Lat.) = Six Ingredients Pill with Rehmannia (Engl.)

In another randomized Chinese study a modified combination of two standard recipes *Bu Yang Huan Wu* and *Liuwei Di Huang* was tested as a decoction. *Liuwei Di Huang* was first described in the Yozheng Zhijue [123]. The mixture of both recipes contains *Astragalus membranaceus radix, Ligustrum lucidum fructus, Paeoniae rubra radix, Lumbricus terrestris, Prunus persicae semen, Rehmanniae viride radix, Corni officinalis fructus, Dioscorea opposita radix; and Alismatis rhizoma, Poria alba, Spatholobi caulis, Scolopendra, Mori fructus, Glycyrrhizae radix, Dipsaci fructus, Lycii fructus, Coicis semen, Atractylodis rhizoma, Phellodendri cortex, Scorpio, Mori ramulus*, and *Cyathula officinalis*.

The remaining dregs of decoction were additionally used for washing the lower extremities. The treatment was used on 32 patients with existing CIPN following different chemotherapies and compared with 32 patients who were treated orally with vitamin B1 2500 ug plus by intramuscular injection with vitamin B1 100 mg. Herbal treatment was found to be significantly more effective to vitamin treatment (*P* < 0.05) [93]. 

#### 3.2.3. Modified Chai Hu Long Gu Mu Li Wan (Chin.) = Modified Saikokaryukotsuboreito (Jap.) = Modified Formula bupleurum cum ostrea et *Fossilia ossis* (Lat.) = Modified Bupleurum, Dragon Bone, and Oyster Shell Formula (Engl.)

*Chai Hu Long Gu Mu Li Wan* is a traditional recipe derived from the *Shang Han Lun* [124]. A modification of this recipe was used in a Chinese randomized trial in which 48 patients with ovarian cancer were examined parallel to chemotherapy with placitaxel. They were divided into a treatment group with paclitaxel alone and a treatment group with paclitaxel plus a combination of oral Chinese herbal decoction treatment and external washing of the feet with Chinese herbs.

The oral combination of herbs consists of *Pseudostellaria heterophylla*, *Pinelliae rhizoma*, *Glycyrrhizae radix*, *Scutellaria baicalensis radix*, *Bupleuri radix*, *Fossilia ossis mastodi*, *Ostreae concha*, *Rubia cordifolia radix*, *Scutellariae barbatae herba,* and *Fritillariae thunbergii bulbi*. The external washing was done with *Astragali membranaceus radix*, *Angelica sinensis radix*, *Paeoniae rubra radix*, *Lumbricus terrestris*, *Ligustici chuanxiong rhizoma*, *Prunus persicae semen,* and *Carthami flos*.

The incidence of CIPN was almost half as high in the patients treated additionally with Chinese herbs as evaluated by clinical testing (*P* < 0.05) [94].

#### 3.2.4. *Geranii herba* plus *Aconiti radix*

External application of a combination of *Geranii herba* and *Aconiti radix* extract has been shown to be effective in a rat model of oxaliplatin evoked neuropathy. Mechanical allodynia and thermal hyperalgesia were alleviated. NGF was increased, substance P decreased in the group treated with *Geranii herba* and *Aconiti radix* extract additionally to oxaliplatin compared to oxaliplatin alone [95].

In the following randomized clinical study 58 patients with CIPN from oxaliplatin, taxol, or capecitabine were assigned prospectively in a controlled randomized trial: 30 patients were assigned to the study group and 28 were used as a control. The clinical study revealed that symptoms of pain, paraesthesia, and swelling were relieved after one week of therapy and it was concluded that *Geranii herba* plus* Aconiti radix* granule can relieve neuropathy and improve the quality of life. Unfortunately the authors did not provide data in the published abstract, which species of *Geranii herba* or *Aconiti radix* they used [95].

#### 3.2.5. Goshajinkigan (Jap.) = Niu Che Sen Qi Wan (Chin.) = Pilula renales plantaginis et achyranthis (Lat.) = Life Preserving Kidney Qi Pill (Engl.)

This formula derives from the *Jisheng Fang*, written by Yan Yonghe, a little known but highly regarded physician of the Song Dynasty, published in 1253 [125]. In Japanese Kampo medicine it is called Goshajinkigan (GJG) and is frequently used as a standardized granule. It contains 10 different herbs (*Rehmannia viride radix, Achyranthis bidentatae radix, Corni fructus, Dioscorea opposita rhizoma, Plantaginis semen, Alismatis rhizoma, Moutan cortex, Cinnamomi cortex, Aconiti lateralis praeparata tuber, *and *Poria alba*) [126]. GJG has antioxidant properties [127, 128].

GJG was tested for its effect on CIPN in animal studies. In a rat model of oxaliplatin-induced neuropathy repeated administration of GJG prevented the oxaliplatin-induced cold hyperalgesia but not mechanical allodynia and axonal degeneration of the rat sciatic nerve. A single administration of GJG reduced both cold hyperalgesia and mechanical allodynia after the development of neuropathy. GJG did not affect the antitumour effect of oxaliplatin on the tumour cells or mice implanted with tumour cells [98].

GJG was also tested in a rat model for paclitaxel-induced peripheral neuropathy, but as with oxaliplatin no regeneration was found in histological examination [96]. Nevertheless further rat animal studies showed a positive effect of GJG on cold allodynia [97].

GJG has been widely used to treat symptoms like numbness, vibration sensation, cold sensation, and limb pain associated with diabetic neuropathy [129].

It has been shown to prevent oxaliplatin-induced peripheral neuropathy in a FOLFOX-regimen (FOL: Folinic acid (leucovorin), F: Fluorouracil (5-FU), OX: Oxaliplatin (Eloxatin)) in clinical studies [99, 100]. In a noncontrolled study 14 patients received GJG every day after the first oxaliplatin infusion. GJG seemed to prevent acute oxaliplatin-induced neurotoxicity [99]. In a retrospective analysis of 45 patients, 22 received GJG during their FOLFOX regimen against nonresectable or recurrent colorectal cancer, while 23 did not get this additional therapy. The incidence of grade 3 PN in the GJG group was significantly lower than in the control group (*P* < 0.01, log-rank test). The incidence of grade 3 PN after 10 courses of chemotherapy was 0% in the GJG group and 12% in the control group, and after 20 courses was 33% in the GJG group and 75% in the control group [100]. A further retrospective analysis was performed in 90 patients who were given a FOLFOX regimen for metastatic colorectal cancer. Two treatment groups were compared: FOLFXOX plus GJG and FOLFOX plus GJG plus Ca^2+^/Mg^+^, and two control groups: FOLFOX without additional therapy and FOLFOX plus Ca^2+^/Mg^+^. When a cumulative dose of oxaliplatin exceeded 500 mg/m^2^, the incidence of PN was 91% in the FOLFOX without additional therapy group, 100% in the FOLFOX group with additional Ca^2+^/Mg^+^ therapy, and 79% in the FOLFOX plus GJG plus Ca^2+^/Mg^+^ therapy group and 50% in the GJG plus FOLFOX group. The cumulative oxaliplatin dose when 50% of patients developed neurotoxicity was 765 mg/m^2^ in the GJG plus FOLFOX and 340 mg/m^2^ in the FOLFOX plus GJG plus Ca^2+^/Mg^+^ group, respectively, and 255 mg/m^2^ in both control groups. The authors concluded that concomitant administration of GJG reduced the neurotoxicity of oxaliplatin without having a negative influence on the response rate [101], so for further validation of these data a concept for a prospective, controlled, double blinded randomized study was developed [130].

GJG was also tested for paclitaxel-induced neuropathy in breast and gynecological cancer. A retrospective study on 82 patients found that GJG was possibly effective for the treatment and the prevention of peripheral neuropathy and seemed to be more effective when administered from the beginning of chemotherapy using paclitaxel [102].

In a prospective study in paclitaxel/carboplatin treated patients with ovarian or endometrial cancer, patients were randomly divided into two groups. 14 patients received vitamin B12 and 15 patients vitamin B12 plus GJG. The observation period was 6 weeks following treatment initiation. A NCI-CTCAE (*National Cancer Institute-Common Toxicity criteria) *grade 3 of neurotoxicity developed in 2 patients (14.3%) after 6 weeks in the vitamin B12, whereas no neurotoxicity was observed in the vitamin B12/GJG group. The change in the frequency of abnormal current perception threshold (CPT) ratio was significantly lower in the VB12/GJG group in comparison to VitB12 alone (*P* < 0.05), which suggests that GJG inhibits the progression of PN [103].

#### 3.2.6. Keishikajutsubuto (Jap.) = Gui Zhi Jia Shu Fu Tang (Chin.) = Decoctum ramulorum cassiae cum atractylodis macrocephae et aconiti (Lat.) = Cinnamon Twig Decoction plus Atractylodes and Aconite (Engl.)

This formula has its basis from the *Shang Han Lun* and was further developed as a Japanese experimental formula during the Edo period (1603 to 1868). It contains *Cinamomi cortex, Aconiti lateralis praeparata tuber, Zingiberis rhizoma, Jujubae fructus, Glycyrrhizae radix, *and* Atracylodis macrocephalae rhizoma*.

This herbal combination with an increased dose of *Aconiti lateralis praeparata tuber* was used as a granule for 11 patients with metastatic colorectal cancer receiving FOLFOX in a noncontrolled study. Reduction of neuropathy was observed in 5 cases after chemotherapy (45.5%) [104]. 

The same herbal granule was also used in a study on postherpetic neuralgia and was found to be effective. In three of 15 patients in this noncontrolled trial continuation of the treatment with *Keishikajutsubuto* was not possible due to hot flashes or gastric discomfort. The remaining 12 patients showed a VAS improvement rate of 76.5 ± 27.7% (mean ± standard deviation) [131].

#### 3.2.7. Ogikeishigomotsuto (Jap.) = Huang Qi Wu Wu Tang (Chin.) = (Decotum quinque medicamentorum cum astragalo (Lat.) = Astragalus and Cinnamon Five Herb Combination (Engl.)

This classical combination derives from *Jin Gui Yao Lue* (Essential Prescriptions from the Golden Chamber) [132]. In Kampo medicine it is called *Ogikeishigomotsuto*, containing *Astragali membranaceus radix, Cinnamomi cortex, Paeonia alba radix, Jujubae fructus, *and* Zingiberis rhizoma. *It has been used in individual cases for neuropathic pain due to ANCA-associated vasculitis [133].

In single case report the granule showed a positive effect on neuropathic pain and it allowed the continuation of the suspended chemotherapy with oxaliplatin [105]. 

#### 3.2.8. Shakuyakukanzoto (Jap.) = Shao Yao Gan Cao Tang (Chin.), Formula glycyrrhizae et paeonia (Lat.), Peony and Licorice Decoction (Engl.)

This classical formula derives from the *Shang Han Lun *[124]. In Kampo medicine it is called *Shakuyakukanzoto* and it is a herbal granule of *Paeonia alba radix *and* Glycyrrhizae radix*. It is used to relieve menstrual pain and muscle spasm as well as muscle pain due to the chemotherapeutic agents paclitaxel and carboplatin [134–136] and has also been tested for CIPN. A retrospective case analysis of 23 patients showed a positive effect on neuropathic pain in CIPN after paclitaxel for ovarian carcinoma [107].

This was supported by animal studies in a mouse model of paclitaxel-induced pain. *Shakuyakukanzoto* significantly relieved the allodynia and hyperalgesia induced by paclitaxel [106]. *Shakuyakukanzoto* has also been tested for preventing neurotoxic side effects of FOLFOX and the effect was retrospectively compared to the treatment with GJG (see Section 3.2.5). 44 patients with metastatic colorectal cancer received FOLFOX and concurrently received either GJG (*n* = 20) or *Shakuyakukanzoto* (*n* = 24). The response was 50.0% in the GJG and 65% in the *Shakuyakkanzoto* group. The authors concluded that both recipes are able to reduce the FOLFOX-induced neurotoxicity [108].

### 3.3. Herbs Tested or Herbal Ingredients for Neuropathic Pain, Not Specifically Tested to CIPN

Capsaicin is the active component of chili peppers, which are plants belonging to the genus *Capsicum*. Topical creams with capsaicin are used to treat peripheral neuropathic pain. Following application to the skin capsaicin causes enhanced sensitivity, followed by a period with reduced sensitivity and, after repeated applications, persistent desensitisation. 

Topical capsaicin is used to treat postherpetic neuralgia and HIV-neuropathy and has been found to be effective in multiple trials. There are risks of epidermal innervation upon repeated application over long periods [137].

*Aconiti lateralis praeparata radix* is a herb used in many recipes for neuropathy like GJG (see Section 3.2.5) and *Keishikajutsubuto* (see Section 3.2.6) and is often used for several types of persistent pain. In a mouse model analgesic effects caused by inhibition of astrocytic activation by *Aconiti lateralis praeparata radix *were mimicked by the intrathecal injection of fluorocitrate. The study indicated that the activation of spinal astrocytes was responsible for the late maintenance phase of neuropathic pain, so *Aconiti lateralis praeparata radix *could be a therapeutic strategy for treating neuropathic pain [138]. In a rat model *Aconiti lateralis praeparata radix *was tested against a placebo for allodynia and thermal hyperalgesia. A dose-dependent effect was measured. The effects were inhibited by intraperitoneal and intrathecal nor-binaltorphimine, a selective kappa-opioid receptor antagonist, but not by intraperitoneal naloxone. The authors concluded that the effect against neuropathic pain is induced via spinal kappa-opioid receptor mechanisms [139].

*Moutan cortex* and *Coicis semen* have been tested positively in two different neuropathic pain mice models. In one model allodynia was induced by intrathecal administration of prostaglandin F2alpha (PGF2alpha) and in the second model by selective L5 spinal nerve transection. The extracts of *Moutan cortex* and *Coicis semen* dose dependently alleviated the PGF2alpha-induced allodynia. The increase in NADPH diaphorase activity in the spinal cord associated with neuropathic pain was also blocked by these extracts. These results suggest that *Moutan cortex* and *Coicis semen *are substances effective in the treatment of neuropathic pain [140].

*Nigella sativa* and one main compound thymoquinone were beneficial on histopathological changes of sciatic nerves in streptozotocin (STZ) induced diabetic rats. Evaluation of the tissues in the diabetic animals showed fewer morphologic alterations, and myelin breakdown decreased significantly after treatment with *Nigella sativa* and thymoquinone. The ultrastructural features of axons also showed improvement [141].

*Ocimum sanctum* was investigated in sciatic nerve transection induced peripheral neuropathy in rats. Axonal degeneration was assessed histopathologically. Paw pressure, Von Frey Hair, tail cold-hyperalgesia, and motor in-coordination tests were performed to assess the in vivo extent of neuropathy. Biochemical estimations of thiobarbituric acid reactive species (TBARS), reduced glutathione (GSH), and total calcium levels were also performed. *Ocimum sanctum* attenuated axonal degeneration, rise in TBARS, total calcium, and decrease in GSH levels in a dose-dependent manner. In vivo reduction of nociceptive threshold and motor in-coordination was found. The authors concluded that anti-oxidant and calcium attenuating actions may be responsible for the amelioration [142].

In an another animal study STZ-induced diabetic rats received intraperitoneal injection of this extract of *Teucrium polium. *The treated rats exhibited a lower nociceptive score as compared to untreated diabetic rats. The results may suggest a therapeutic potential of *Teucrium polium* for the treatment of hyperalgesia [143].

A hexanic extract from *Phyllanthus amarus *has been shown to be effective in a neuropathic model of nociception. The antiallodynic effects seemed not to be associated with the impairment of motor coordination or with the development of tolerance. Apart from its anti-inflammatory actions, which are probably linked to the presence of lignans, another as yet unidentified active principle(s) present in the hexanic extract of *Phyllanthus amarus* produces pronounced anti-allodynia [144]. 

 An aqueous extract of *Sesbania sesban* was tested in STZ-induced diabetic rats. The treated group showed an increased tail flick latency significantly when compared with pregabalin and reduced superoxide anion and total calcium levels which gave evidence of neuroprotective effects [145].

## 4. Discussion

In spite of intense research in the last decades, no conventional pharmacological substance has been established as a sufficient and safe treatment of CIPN-induced neuroprotection and regeneration [31, 43–63, 65–67]. Herbal treatment is commonly used for different kind of therapies where western medicine does not offer a sufficient efficacy, but the evidence of the use of herbal treatment is not clear and has to be elucidated. 

One main problem of doing research on herbs and understanding the mechanisms of their action is the fact that herbs contain a number of active compounds and by tradition, especially in Asian herbal therapy, combinations of multiple herbs are common. 

Classical research mainly focusing on a single active compound has been done often without regard to historical knowledge of the therapeutic utility of the plant source [146]. This does not reflect the complexity of traditional Asian herbal recipes, while there is some evidence that single components extracted from plants are less potent than the complete extract [147] and a multitarget approach might be more effective than a single target approach [148]. Pharmacological mixtures can also have the advantage of potentiating the action of their multiple bioactive components and the option of an individualized therapy [149, 150]. For scientific understanding of the mechanisms of action there is still a need for basic research on single herbs and their active compounds, but research should not stop at this level but continue with research of multicompounds, their interactions, and increasing or decreasing activity in combinations.

The positive effect of a single herb on neuroprotection or regeneration was not found in any clinical study and rarely found in animal studies. Only the flavonoid apigenin from *Salvia officinalis* seems to protect neurons against toxicity [116] and *Ginkgo biloba* as a single herb shows some evidence of preventing neuronal degeneration and inducing neural regeneration in CIPN [109–111].

Mainly in animal studies only a few single herbs have been tested for treatment of CIPN improving neuropathic pain. This effect might be induced by *Acorus calamus rhizoma* due to its antioxidative, anti-inflammatory, and calcium inhibitory actions [70]. Flavonoids from *Matricaria chamomilla* have an antispasmodic and an anti-inflammatory effect [83, 84]. *Salvia officinalis* is probably working due to its antioxidant activity and anti-inflammatory properties [112–115] and is also involved in an opioid mechanism [119]. *Fritillaria bulbus* might be effective due to its isosteroidal alkaloid verticinone [91]. While *Cannabis sativa* is effective in multiple and central pain syndromes [72–78], the fact that two *Cannabis* agonists had been shown to be effective in CIPN in a rat model could be a hint that *Cannabis sativa* might be a promising substance for pain in CIPN in the future. 

Only *Ginkgo biloba* extract has been positively tested in a small retrospective clinical study in humans [87] and *Sweet bee venom* had shown positive results by injection into acupuncture points in a small clinical case series [89, 90]. Topical capsaicin from chili peppers was found to be effective in multiple trials to treat pain from postherpetic neuralgia and HIV-neuropathy but has not been specifically tested against pain in CIPN [137].

There are a few more single herbs tested in animal models for the treatment of neuropathic pain in other conditions than CIPN. *Moutan cortex* and *Coicis semen* have been tested positively in two different neuropathic pain models. Both herbs dose dependently alleviated the PGF2alpha-induced allodynia and blocked NADPH diaphorase activity in the spinal cord associated with neuropathic pain [140]. 

*Nigella sativa*, *Ocimum sanctum*, *Teucrium polium*, *Phyllanthus amarus,* and *Sesbania sesban* also have been tested in single studies to ameliorate neuropathic pain [141–145].

*Aconiti lateralis praeparata radix* in a mouse model of CIPN had analgesic effects by inhibition of spinal astrocytic activation [138] and in a rat model *Aconiti lateralis praeparata radix *had a dose-dependent effect on allodynia and thermal hyperalgesia which was induced via spinal kappa-opioid receptor mechanisms [139], which confirmed similar animal studies on neuropathic pain in diabetic mice [148]. Active components are alkaloids, for example, aconitine and mesaconitine and have in addition pain-relieving effects, cardiotonic and vasodilator actions [151–155].

*Aconiti lateralis praeparata radix* is often used for several types of persistent pain while it is rarely used as a single herb for CIPN. But interestingly *Aconiti lateralis praeparata radix* is part of many recipes used for CIPN as GJG, *Shakuyakukanzoto, Keishikajutsubuto,* and *Geranii herba *plus* Aconiti radix* combination (see Sections 3.2.4, 3.2.5, 3.2.6, and 3.2.8).

Some of the pain-relieving effects of GJG are known to be induced by aconite [139] and it is considered that the analgesic effect is exerted by the suppression of pain-transmitting substances release by *κ*-opioid receptor stimulation mediated by dynorphin, an endogenous opioid substance released by processed aconite [148]. But on the other hand, these effects were stronger, when using of the full recipe in comparison to the use of *Aconiti lateralis praeparata radix *alone [155].

GJG is the best tested herbal recipe for CIPN. But not all details of its mechanism have been clearly identified and for some ingredients the effects are unknown. It is considered that the analgesic effect is exerted through the improvement of peripheral nocireceptor sensitivity, vasodilation, and peripheral circulation by the promotion of NO production due to the effects of *Alismatis rhizoma* and *Dioscorea opposita rhizoma* mediated by the bradykinin B2 receptor and the muscarinic acetylcholine receptors [148]. Another substance from GJG, *Rehmanniae praeparata radix,* could promote the function of learning and memory of MSG rats, and its mechanism may be related to the increase of the expression of hippocampal c-fos and nerve growth factor (NGF) [156], which can be relevant for regeneration in CIPN as well, while NGF exhibits potent biological activities such as preventing neuronal death, promoting neurite outgrowth, and supporting synapse formation [157]. One further mechanism of GJG might be its positive effect on improving the microcirculation, which might be helpful for the recovery of the nerves in CIPN [158].

Even though the full recipe was clinically positively tested in a couple of clinical trials, most of them were uncontrolled or retrospective analyses [99–102, 130] and the only prospective trial had a limited number of participants [103]. So the evidence of a positive effect is still low and needs further clinical and experimental confirmation.

GJG has also been compared to another herbal recipe, *Shakuyakukanzoto,* and both were found to be effective in the treatment of CIPN, but there was no negative control group [107]. *Shakuyakukanzoto* was analyzed in a mouse model [106] and in a retrospective analysis of 23 cases without controls [108]. Since *Shakuyakukanzoto* contains only two herbs, *Paeonia alba radix* and *Glycyrrhizae radix,* the future evaluation of its role for treating CIPN might be easier than for other herbal recipes. While neuronal apoptosis can be triggered by oxidative stress and mitochondrial dysfunction, substances with an antioxidative potency are possible candidates for treatment of CIPN [19–22], so the fact that paeoniflorin as one bioactive component of *Paeonia alba* radix has been positively tested as an antioxidant in a nonneuronal cell model might be relevant [159]. It has also neuroprotective effects which are closely correlated to activating the adenosine A1 receptor, ameliorating the function of the cholinergic nerve, regulating ion channel homeostasis, retarding oxidative stress, apoptosis of the neurocytes, and promoting nerve growth [160]. *Paeonia alba radix* was used in another recipe (see Section 3.2.7) and in two recipes *Paeonia rubra radix* (see Sections 3.2.1 and 3.2.2) has been used. *Paeonia alba radix* and *Paeonia rubra radix* have a lot of similarities in their components and paeoniflorin is a bioactive compound of both herbs [161].

While the reason for using herbal recipes derives from historical knowledge or transferral of historical concepts to modern diseases, interestingly four of eight herbal recipes tested for CIPN contain *Glycyrrhizae radix*. To find the scientific ratio behind this decision, analyses of its action in the context of neural cell damage might be fruitful.

Another herb that has been used in four of eight herbal recipes tested for CIPN is *Astragalus membranaceus radix*. This might be rational, while *Astragalus membranaceus radix* water extracts caused a marked enhancement of the NGF-mediated neurite outgrowth and the expression of growth-associated protein 43 from PC12 cells in vitro [162]. *Astragalus membranaceus radix* extracts can be a potential nerve growth-promoting factor, being salutary in aiding the growth of axons in the peripheral nerve [162]. Astragaloside IV (AGS-IV), one bioactive compound of *Astragalus membranaceus radix*, is an aldose-reductase inhibitor and a free-radical scavenger which suppressed a decrease in myelinated fibers, promoted an increase in myelinated fiber density and an increase in segmental demyelination in diabetic rats [163]. It also increased the activity of glutathione peroxidase in nerves, depressed the activation of aldose reductase in erythrocytes, decreased the accumulation of advanced glycation end products in both nerves and erythrocytes, and elevated Na+, K+-ATPase activity in both the nerves and erythrocytes of diabetic rats, so it is considered to be protective against the progression of peripheral neuropathy [163].

The herbal recipe *Bu Yang Huan* and a modified extension have been used in two clinical controlled randomized trials [92, 93] (3.2.1 and 3.2.2, see above) and was found to be effective in both. The formula used is very complex, and little is known about the mechanism of its compound, but herbs like *Astragalus membranaceus radix, Paeonia rubra radix, *and *Dioscorea opposita rhizoma* are part of the herbal combination where possible mechanisms are described (3.2.1, 3.2.2, 3.2.5, 3.2.7, see above), so at least something is known about its possible mechanisms of effect.

Another study used a modified classical prescription named *Chai Hu Long Gu Mu Li Wan* (see Section 3.2.3) and found positive results as well [94]. Even though it is a controlled randomized trial, the treatment protocol is very complicated, combining oral intake of medical herbs and external washing with other herbs. So also with this herbal combination little is known about the mechanism of its action. 

Two other herbal recipes have been reported for successful clinical use, *Keishikajutsubuto* = *Gui Zhi Jia Shu Fu Tang* (see Section 3.2.6) and Ogikeishigomotsuto = *Huang Qi Wu Wu Tang* (see Section 3.2.7). But these studies are either uncontrolled or a case report, so the quality of the evidence is very low.

The trials on the single herbs and herbal combinations tested so far do not provide a clear recommendation for clinical use. But research on some single herbs as well as on combinations like GJG is promising, even though the level of knowledge is limited to basic research on the mechanisms of action and evidence from clinical trials. But it is necessary to continue research on this field, while treatment concepts for CIPN are lacking. 

The most used herbs of herbal recipes in clinical trials on CIPN are *Aconiti lateralis praeparata radix*, *Rehmannia praeparata radix*, *Paeonia (alba* and *rubra) radix*, *Astragalus membranaceus radix,* and *Glycyrrhizae radix*. 

In Chinese medicine experience *Astragalus membranaceus radix* is supporting the Qi, which means in western terms supporting the general energy level of the body, which is usually reduced in CIPN by activating the vegetative nervous system. *Rehmannia praeparata radix* is basically supporting the Yin, which in western terms reflects the structural damage of tissue, in the case of CIPN the peripheral nerves. *Paeonia alba radix* and *Paeonia rubra radix* promote the flow of the Xue, which in western terminology has its correlate in enhancing the microcirculation which might be reduced in CIPN; promotion of the perfusion might enhance the regeneration. 

*Aconiti lateralis praeparata radix* supports in Chinese medicine theory the Yang. A typical symptom of Yang deficiency is ice-coldness, which is prominent in the extremities in some cases of CIPN. *Glycyrrhizae radix* supports the fluids and has a balancing effect on the whole recipe.

So in spite of using different terms, Chinese medicine theory describes physical reactions of the body that can be explained by western physiology and has found its proof by experimental studies [138, 139, 148, 151–156, 159–164].

So using these herbs has as rational foundation on the basis of Chinese medical theory as well as from experimental studies, but unfortunately still little is known about the complex physiological mechanisms of herbal combinations, the interactions of the substances, and the mechanisms of action of many other substances used in herbal medicine. The challenge for future research is bringing historical knowledge and modern scientific analysis together. 

Due to this limited knowledge on the mechanisms of the action of herbs, we additionally collected data on herbs that are a putative treatment option for CIPN. While oxidative stress and mitochondrial dysfunction promote CIPN, substances with an antioxidative potency are possible candidates for the treatment of CIPN. So in the list below we list herbal antioxidants tested in neuronal cell or disease models. Additionally we added herbs for enhancement of nerve growth as putative treatment options for CIPN. NGF exhibits potent biological activities such as preventing neuronal death, promoting neurite outgrowth, and supporting synapse formation [157], which has a relevance for the development of CIPN [39].

*Putative Herbs or Herbal Compounds for the Treatment of CIPN*

1. *Herbal Antioxidants Tested in Neuronal Cell or Disease Models.* Puerarin (from *Pueraria lobata radix*) [165, 166], Icariin (from Epimedii herba) [167], a fraction of polysaccharides (from *Lycium barbarum*) [168], Ginsenoside Rg1 (from Notoginseng panacis) [169], honokiol and magnolol (from *Magnolia officinalis*) [170], Ginkgolide A, B (from *Ginkgo biloba*) [171], huperzine A (from *Huperzia serrata*) [171], Ginseng radix [172], Notoginseng panacis radix [173], 3,5,4′-tetrahydroxystilbene-2-O-beta-D-glucoside (from *Polygonum multiflorum*) [174–176], celastrol (from *Tripterygium wilfordii* Hook) [177], Salvianolic acid B (from *Salvia miltiorrhiza*) [178, 179], tanshinone IIA (from *Salvia miltiorrhizae*) [180], *Gastrodia elata* rhizoma [181, 182], Astragaloside IV (from *Astragalus membranaceus radix*) [163], Tetramethylpyrazine (from *Ligusticum wallich*i) [183, 184], Ziziphus spinosus semen [185], Baicalein (from *Scutellaria baicalensis radix* Georgi) [186], *Uncaria rhynchophylla* [164], 6,7-dihydroxy-2-methoxy-1,4-phenanthrenedione, chrysoeriol 4′-O-beta-D-glucopyranoside, chrysoeriol 7-O-beta-D-glucopyranoside, and alternanthin (from *Dioscorea opposita radix*) [187].
2. *Herbs or Herbal Compounds That Enhance Nerve Growth*. Cistanches herba [157, 188], Huperzine A (HupA) from *Huperzia serrata* [189, 190], a lipophilic fraction of panax ginseng [191]. *Astragalus membranaceus radix* [162], Gentiside C, a compound of *Gentiana rigescens radix* [191], *Rehmanniae praeparata radix* [192], *Paeoniflorin* of *Paeonia alba radix* [193].

Research with the aim of proving the benefits of promising substances or herbal combinations has to describe the mechanism of action for single herbs and single components, investigate the interactions of combinations of substances, and analyse the promoting effects of combinations.

From this viewpoint, research in this field has not very much progressed, but if research does not provide these data, herbal combinations will not find acceptance in mainstream treatments in non-Asian countries in Europe, North America, or Australia. 

This challenge could be taken if more international cooperation of interested research groups would be organized. 

## 5. Conclusion

Experimental and clinical studies have not yielded enough evidence to establish a standard practice for the treatment of CIPN, but from this literature review, a lot of promising substances, mainly Chinese medical herbs with possible effect in CIPN or a putative influence on mechanisms of CIPN, have been identified in the last years. 

The knowledge of the mechanisms of action is still limited and the quality of the clinical trials needs further improvement. In the future not only the mechanisms of action for single herbs and single components have to be described, but interactions of combinations of substances as well as interactions with chemotherapy have to be investigated and analysed in depth. While CIPN has multiple possible mechanisms of neuronal degeneration, a combination of components might be a promising opportunity focusing on multiple targets of degeneration or activating regeneration.

## Figures and Tables

**Table 1:** Medical herbs tested for CIPN.

Section no.	Medical herb(s)	Study design	Chemotherapy	Outcome	Author/year
Single herbs or single compounds					
3.1.1	*Acorus calamus rhizoma* (hydroalcoholic extract)	Rat model	Vincristine	Improvement of neuropathic pain	Muthuraman and Singh, 2011 [70]
3.1.2	*Cannabis*-Receptor agonists, R-AM1241 and Am-1714	Rat model	Paclitaxel	Allodynia, antinociceptive	Rahn et al., 2008 [71] multiple trials for neuropathic pain not due to chemotherapy, for example, Ellis et al. [72], Karst et al. [73], Ware et al. [74], Rog et al. [75], Blake et al. [76], Berman et al. [77], Wilsey et al. [78] with Cannabis sativa
3.1.3	*Matricariachamomilla* (hydroalcoholic extract)	Mouse model	Cisplatin	Improvement of neuropathic pain	Abad et al. 2011 [85]
3.1.4	*Ginkgo biloba* (alcoholic extract)	(1) Mouse model (2) Retrospective study (*n* = 17)	Oxaliplatin	(1) Neuroprotection (2) Improvement of neuropathic pain	(1) Oztürk et al., 2004 [86] (2) Marshall et al., 2004 [87]
3.1.5	*Salvia officinalis* (hydroalcoholic extract)	Mouse model	Vincristine	Improvement of neuropathic pain	Abad et al., 2011 [88]
3.1.6	*Sweet bee venom* (pharmacopuncture)	(1) Case series (*n* = 5) (2) Case series (*n* = 11)	Taxol, paclitaxel, or carboplatin	Injection into acupoint (Zusanli, St 36) (1) Effect on pain and neuropathy scales (2) Effect on VAS and HRQOL scores	(1) Park et al., 2011 [89] (2) Yoon et al., 2012 [90]
3.1.7	Verticinone, hydroalcoholic extracted from *Fritillaria bulbus *	Mouse model and rat model	Paclitaxel	Inflammatory and neuropathic pain, dose dependent, no tolerance	Xu et al., 2011 [91]

Herbal combinations					
3.2.1	*Bu Yang Huan Wu * ^∗1^ (*decoction*)	Randomized trial, *n* = 44, control = 40	Oxaliplatin	Improvement of clinical scales	Sun et al., 2008 [92]
3.2.2	modified *Bu Yang Huan Wu* plus *Liuwei Di Huang * ^∗2^ (*decoction*)	Randomized trial, *n* = 32, control Vit. B1 = 32	Different chemotherapies	Improvement of clinical scales	Deng and Zou, 2007 [93]
3.2.3	modified *Chai Hu Long Gu Mu Li Wan * ^∗3^ (*decoction*)	Randomized trial, *n* = 26, control = 22	Paclitaxel	neuroprotection	Pan et al., 2012 [94]
3.2.4	*Geranii herba* plus Aconiti radix (granule)	(1) Rat model (2) Randomized, prospective trial *n* = 30, control = 28	(1) Oxaliplatin (2) Oxaliplatin, taxol or capecitabine	(1) Allodynia (2) Neuropathic pain, paraesthesia, selling. external washing	Sima and Pan, 2009 [95]
3.2.5	Goshajinkigan = *Niu Che Sen Qi Wan * ^∗4^ (granule)	(1) Rat model (2) Rat/mouse model	(1) Paclitaxel (2) Oxaliplatin	(1) No neurogeneration, improvement of allodynia (2) Improvement of neuropathic pain, no neuroregeneration	(1) Hashimoto et al., 2004 + 2006 [96, 97] (2) Ushio et al., 2012 [98]
		(1) Noncontrolled study *n* = 14 (2) Retrospective study GJG = 22, control = 23 (3) Retrospective study GJG = 45, control = 45 (4) Retrospective study *n* = 82 (5) Randomized prospective study Vit B12 = 14, VitB12/GJG = 15	(1) Oxaliplatin (2) Folfox^∗8^ (3) Folfox^∗8^ (4) Paclitaxel (5) Paclitaxel/ carboplatin	(1) Reduced acute neurotoxicity (2) Neuroprotection (3) Neuroprotection No change of anticancer activity (4) Neuroprotection, better when administered early (5) Less severe neurotoxicity, better CPT in GJG group	(1) Shindo et al., 2008 [99] (2) Nishioka et al., 2011 [100] (3) Kono et al., 2011 [101] (4)Yamamoto et al., 2009 [102] (5) Kaku et al., 2012 [103]
3.2.6	Keishikajutsubuto = Gui Zhi Jia Shu Fu Tang^∗5^ (granule)	Uncontrolled study *n* = 15 (3 dropouts)	Folfox^∗8^	76.6% mean improvement on VAS	Yamada et al., 2012 [104]
3.2.7	Ogikeishigomotsuto = Huang Qi Wu Wu Tang^∗6^ (granule)	Case report *n* = 1	Oxaliplatin	neuropathic pain	Tatsumi et al., 2009 [105]
3.2.8	*Shakuyakukanzoto* = Shao Yao Gan Cao Tang^∗7^ (granule)	(1) Mouse model (2) Retrospective case analysis *n* = 23 (3) Retrospective clinical study, comparison GJG = 20, SYK = 24	(1) and (2) paclitaxel (3) Folfox^∗8^	(1) Allodynia, hyperalgesia (2) Effect on neuropathic pain (3) 50% response in Shakuyu-kanzoto and 65% in Goshajinkigan on prevention of neurotoxicity	(1) Hidaka et al., 2009 [106] (2) Fujii et al., 2004 [107] (3) Hosokawa et al., 2012 [108]

^∗1^ *Astragalus membranaceus radix, Angelica sinensis radix, Prunus persicae semen, Paeoniae rubra radix, Ligustici chuanxiong rhizoma, Lumbricus terrestris, Spatholobi caulis, Curcuma radix, Chaenomeles lagenaria fructus*, and *Achyranthes bidentata*.

^∗2^ *Astragalus membranaceus radix, Ligustrum lucidum fructus, Paeoniae rubra radix, Lumbricus terrestris, Prunus persicae semen, Rehmanniae viridae radix, Corni officinalis fructus, Dioscorea opposita* radix; and *Alismatis rhizoma, Poria alba, Spatholobi caulis, Scolopendra, Mori fructus, Glycyrrhizae Radix, Dipsaci fructus, Lycii fructus, Coicis semen, Atractylodis rhizoma, Phellodendri cortex, Scorpio, Mori ramulus*, and *Cyathula officinalis*.

^∗3^ *Pseudostellaria heterophylla, Pinelliae rhizoma, Glycyrrhizae radix, Scutellaria baicalensis radix, Bupleuri radix*, *Fossilia ossis mastodia draconis*, *Ostrae concha*, *Rubiae cordifoliae radix, Scutellariae barbatae herba*, and* Fritillaria thunbergii bulbi. *The external washing was done with* Astragali radix, Angelica sinensis radix, Paeoniae rubra radix, Lumbricus terrestris, Ligustici chuanxiong rhizoma*, *Prunica persicae semen*, and *Carthami flos*.

^∗4^ *Rehmannia viride radix, Achyranthis bidentatae radix, Corni fructus, Dioscoreaopposita rhizome, Plantaginis semen, Alismatis rhizoma, Moutan cortex, Cinnamomi cortex, Aconiti lateralis praeparata tuber*, and *Poria alba*.

^∗5^ *Cinnamomi cortex, Aconiti lateralis praeparata tuber, Zingiberis rhizoma, Jujubae fructus, Glycyrrhizae radix*, and *Atracylodis macrocephalae rhizoma*.

^∗6^ *Astragali membranaceus radix, Cinnamomi cortex, Paeonia alba radix, Jujubae fructus*, and* Zingiberis rhizoma*.

^∗7^ *Paeonia alba radix* and* Glycyrrhizae radix*.

^∗8^Chemotherapeutic regime with FOL: Folinic acid (leucovorin), F: Fluorouracil (5-FU), and OX: Oxaliplatin (Eloxatin).
